# Beyond the passive interactions at the nano-bio interface: evidence of Cu metalloprotein-driven oxidative dissolution of silver nanoparticles

**DOI:** 10.1186/s12951-016-0160-6

**Published:** 2016-01-22

**Authors:** Daniel N. Freitas, Andrew J. Martinolich, Zoe N. Amaris, Korin E. Wheeler

**Affiliations:** Department of Chemistry and Biochemistry, Santa Clara University, Santa Clara, CA 95053 USA; Department of Chemistry, Colorado State University, Fort Collins, CO 80523-1872 USA

**Keywords:** Nanomaterials, Nanoparticles, Silver nanoparticles, Protein corona, Redox chemistry, Cu metalloprotein, Azurin

## Abstract

**Background:**

In a biological system, an engineered nanomaterial (ENM) surface is altered by adsorbed proteins that modify ENM fate and toxicity. Thus far, protein corona characterizations have focused on protein adsorption, interaction strength, and downstream impacts on cell interactions. Given previous reports of Ag ENM disruption of Cu trafficking, this study focuses on Ag ENM interactions with a model Cu metalloprotein, Cu(II) azurin. The study provides evidence of otherwise overlooked ENM-protein chemical reactivity within the corona: redox activity.

**Results:**

Citrate-coated Ag ENMs of various sizes (10–40 nm) reacted with Cu(II) azurin resulted in an order of magnitude more dissolved ionic silver (Ag(I)(*aq*)) than samples of Ag ENMs only, ENMs mixed Cu(II) ions, or control proteins such as cytochrome c and horse radish peroxidase. This dramatic increase in ENM oxidative dissolution was observed even when Cu(II) azurin was combined with a diverse mixture of *Escherchia coli* proteins to mimic the complexity of the cellular conona. SDS PAGE results confirm that the multiprotein ENM corona includes azurin. A Cu(I)(*aq*) colorimetric indicator confirms Cu(II) azurin reduction upon interaction with Ag ENMs, but not with the addition of ionic silver, Ag(I)(*aq*).

**Conclusions:**

Cu(II) azurin and 10–40 nm Ag ENMs react to catalyze Ag ENM oxidative dissolution and reduction of the model Cu metalloprotein. Results push the current evaluation of protein-ENM characterization beyond passive binding interactions and enable the proposal of a mechanism for reactivity between a model Cu metalloprotein and Ag ENMs.

**Electronic supplementary material:**

The online version of this article (doi:10.1186/s12951-016-0160-6) contains supplementary material, which is available to authorized users.

## Findings

### Background

The design of metal and metal oxide engineered nanomaterials (ENMs) are complicated by chemical and physical changes in a physiological system. Protein adsorption, for example, forms a “corona” and leaves ENM surfaces with little resemblance to the original material [1–3] to permanently alter ENM reactivity [2, 4–8]. Current ENM protein corona studies focus on characterization of protein adsorptions [9–11] and mediation of downstream biological uptake and toxicity [5, 6, 12, 13]. Proteins and metal ENMs are, however, likely to undergo chemical reactions and alter biological and environmental reactivity of ENMs. Despite the evidence that ENMs are biochemically reactive [14–16] and some even demonstrate enzyme-like activity [16], characterization of ENMs chemically reacting with proteins (beyond protein binding and unfolding) has been mostly overlooked.

With this in mind, Cu metalloproteins were chosen as a case study characterization of protein-ENM biochemical reactivity because recent studies by Armstrong et al. [17] and others [18] have demonstrated that Ag ENMs can disrupt copper trafficking. Importantly, free silver ions (Ag(I)(*aq*)), by contrast, were found to have no impact on Cu trafficking. Results suggest that Cu-metalloenzymes react uniquely with Ag ENMs within the organisms studied and establish the need to specifically evaluate the biochemical interactions between Cu metalloproteins and Ag ENMs.

Cu(II) azurin, a model Cu metalloprotein, is extensively characterized, redox active, and structurally simple with one metal center [19]. Importantly, previous studies of Cu(II) azurin–Ag ENM interactions align with findings by Armstrong et al. [17]; when directly interacting with the Ag ENM surface Cu(II) azurin forms biologically inactive, but fully folded apo- and Ag(I) azurin [20]. Ag(I)(*aq*), however, cannot displace the tightly bound Cu(II) within azurin, a result consistent with previous work [21]. The reactivity of Cu(II) azurin with Ag ENMs, but not with Ag(I)(*aq*), justifies further study in the elucidation of biochemical reactivity between Cu metalloproteins and Ag ENMs.

Here, we evaluate the hypothesis that Cu(II) azurin–Ag ENM are a redox pair. Evidence is presented for Cu(II) azurin binding to Ag ENMs and increasing oxidative dissolution to form Ag(I)(*aq*), even in the presence of a complex mixture of other bacterial proteins. Evidence is also presented for reduction of Cu(II) azurin. Taken together, these experiments explain the unique behavior of Ag ENMs with Cu(II) azurin and, by extension, provide biochemical reactivity as a foundation for Ag ENM modification of Cu homeostasis. More broadly, these data introduce the importance of considering the biochemical reactivity of ENMs beyond passive binding interactions.

### Methods

#### Sample preparation

Citrate-coated ENMs were purchased from Nanocomposix Inc (San Diego, CA). Cu(II) azurin was overexpressed and purified as previously described [20, 22]. Other chemicals were purchased from Fisher Scientific, unless otherwise noted. Eppendorf Centrifuge 5424 and Molecular Devices SpectraMax M2 were used for centrifugation and UV–Vis spectra, respectively.

Unless otherwise stated, all samples included 50 μM Cu(II) azurin, horseradish peroxidase (HRP, Sigma), or equine cytochrome c (cyt c, US Biologicals) reacted with Ag ENMs in nanopure water (18 mΩ). ENM concentrations ensured equal surface area and protein binding sites [23] at 3.73, 0.955, 0.416 and 0.233 nM for 10, 20, 30, and 40 nm ENMs, respectively. Soluble protein extract (SPE) was taken from *E. coli Migula Castellani* and *Chalmers* (ATCC) and used at 0.7 and 0.07 mg/ml, which is equivalent to 50 and 5 µM azurin. Procedures for SPE extraction and purification are provided in supplemental materials.

#### ICP-MS quantification of Ag ENM dissolution

Samples were centrifuged (30 min, 21 K RCF) to remove ENMs from solution after 6 h incubation. 85 % of the supernatant was removed and re-centrifuged. Again, 85 % of the supernatant was removed for preparation and analysis of Ag(I) concentration using an Agilent 7500CE ICP-MS (Agilent Technologies, Palo Alto, CA, USA) by the Interdisciplinary Center for Plasma Mass Spectrometry (University of California at Davis, CA, USA). The samples were introduced using a MicroMist Nebulizer (Glass Expansion, Pocasset, MA, USA) into a temperature-controlled spray chamber. Instrument standards diluted from Certiprep 2A (SPEX CertiPrep, Metuchen, NJ, USA) encompassed the range 0, 0.5, 1, 10, 50, 100, 200, 500, 1000 parts per billion (ppb) in 3 % trace element grade HNO_3_ (Fisher Scientific, Fair Lawn, NJ, USA) in 18.2-MΩ water. A separate 100 ppb Certiprep 2A standard was analyzed as every tenth sample as a quality control. Sc, Y and Bi Certiprep standards (SPEX CertiPrep) were diluted to 100 ppb in 3 % HNO_3_ and introduced by peripump as an internal standard.

#### BCA analysis for Cu(I) detection

The reactions for Cu(I) detection were executed as described above. After 6 h, 100 µL of each sample was combined with 1 mM bicinchoninic acid (BCA, MP Biomedicals, LLC) and analyzed immediately via UV–Vis spectrophotometry using a Shimadzu UV-1800 for Cu(I) detection at 562 nm (ε_562_ = 14,150 M^−1^ cm^−1^). To enable peak analysis, ENMs were centrifuged out of solution (30 min, 15 K RPM) and the supernatant was analyzed for Cu(I) using BCA. To ensure BCA was not reacting with Cu(II) from Cu(II) azurin, Cu(II)(*aq*), or Ag(I)(*aq*), an array of controls were also run as described in supplemental materials.

### Results and discussion

To assess the role of proteins in catalysis of oxidative dissolution, Ag ENM oxidative dissolution was measured across 10-40 nm ENMs with the addition of Cu(II) azurin and two other well-characterized, redox active proteins: heme-containing proteins cytochrome c (cyt c) and horse radish peroxidase (HRP). Cu(II) azurin was also mixed with SPE in varying concentrations to evaluate catalysis of Ag ENM oxidative dissolution within a complex protein mixture like that in the cell. Independent evidence of redox activity was measured through quantification of Cu(II) azurin reduction and Cu(I) release. With multiple sources of evidence for Ag ENM- Cu(II) azurin redox activity, a mechanism of Ag ENM-Cu(II) azurin reactivity is proposed.

#### Cu(II) azurin catalyzes Ag ENM dissolution

**Fig. 1:**
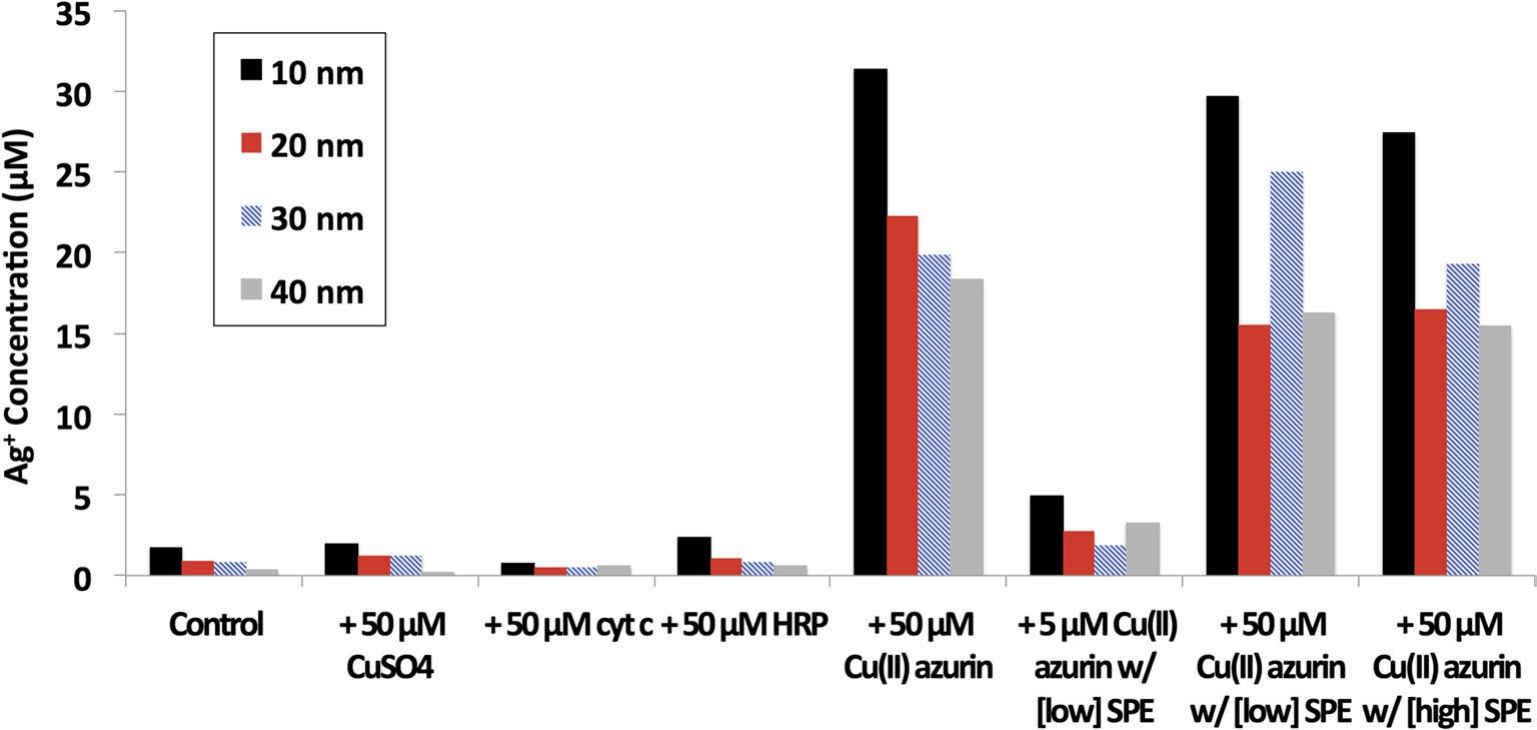
ICP-MS measurements of Ag(I)(*aq*) concentrations from Ag ENM oxidative dissolution. Oxidative dissolution of Ag ENMs was compared across four sizes: 10 (*black*), 20 (*red*), 30 (*blue*), and 40 nm (*grey*). Dissolution of Ag ENMs alone (labeled control) is compared to dissolution in the presence of 50 µM copper sulfate, cyt c, HRP, and Cu(II) azurin. In addition, both 5 and 50 µM Cu(II) azurin with 0.07 mg/ml SPE were reacted with Ag ENMs (labeled [low] SPE), as well as a higher concentration sample with 50 µM Cu(II) azurin with 0.7 mg/ml SPE (labeled [high] SPE). Data for samples with SPE present in solution are shown with the contribution of SPE subtracted from the total Ag(I)(*aq*) concentration. Raw data for samples with SPE are given in Additional file 1: Figure S1

Formation of Ag(I)(*aq*) by oxidative dissolution of 10–40 nm Ag ENMs was quantified by ICP-MS (Fig. 1). Oxidative dissolution was small at 2 µM Ag(I)(*aq*) or less for Ag ENMs alone, or with CuSO_4_, cyt c, and HRP. Addition of Cu(II) azurin, however, increased Ag(I)(*aq*) concentrations by roughly an order of magnitude. Protein driven oxidative dissolution has been previously reported for Ag ENMs, as well as other metal and metal oxide ENMs.

When azurin was mixed with varying concentrations of SPE, Cu(II) azurin still catalyzed Ag ENM dissolution. When the contribution of SPE is removed, Ag(I)(*aq*) concentrations are similar to those found in samples of ENM and azurin alone (Fig. 1, see Additional file 1: Figure S2 for raw concentrations). Even at lower, 5 μM azurin concentrations, where azurin does not dominate the protein corona (SDS PAGE results, Additional file 1: Figure S3) oxidative dissolution is two to four times that measured in control samples. SDS PAGE gels indicate that Cu(II) azurin is within the hard corona even when mixed with SPE (Additional file 1: Figure S3).

Notably, although Ag ENM reduction potentials are size dependent, there is not a clear size dependence in Ag ENM oxidative dissolution within this sample set; the sole exception is that highest dissolved Ag(I)(*aq*) concentrations were consistently observed in the smallest, 10 nm ENM samples.

#### Ag ENMs reduce Cu(II) azurin

**Fig. 2:**
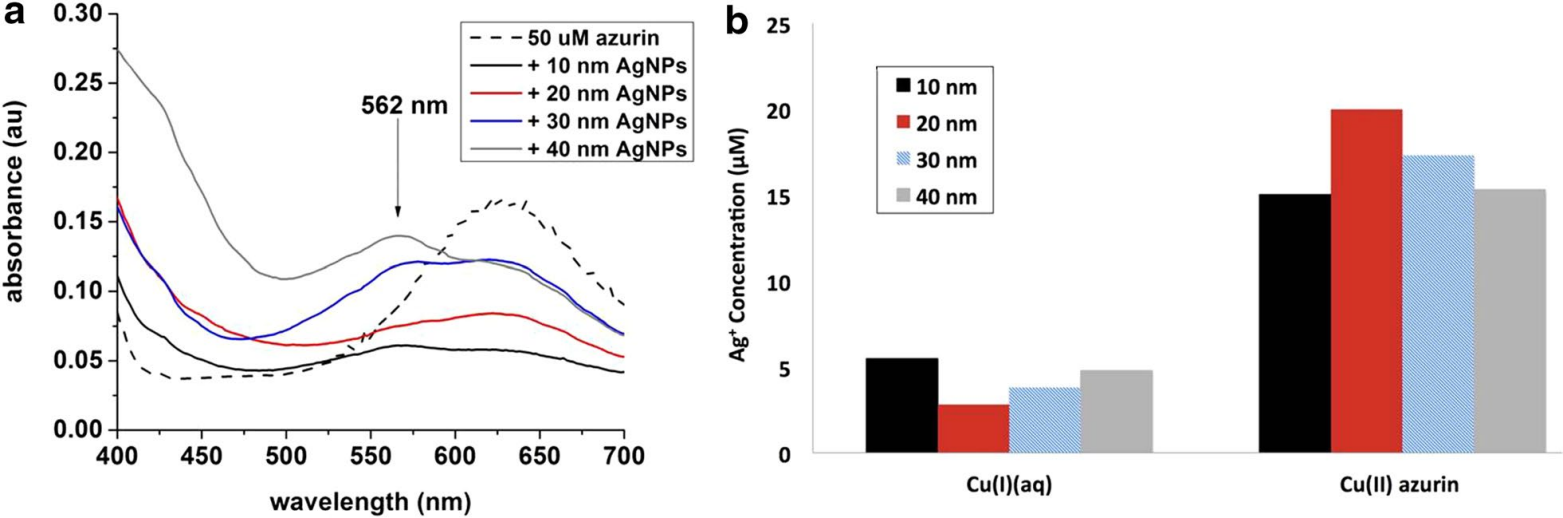
Colorimetric BCA assay of Cu(I)(*aq*) concentrations. **a** Spectra of BCA added to 50 µM Cu(II) azurin (*black dashes*) and added to a reacted mixture of 50 µM Cu(II) azurin with 10 (*black*), 20 (*red*), 30 (*blue*), and 40 nm Ag ENMs (*grey*). The BCA-Cu(I) complex (λmax = 562 nm) presents as a shoulder on the Cu(II)-thiol LMCT band from Cu(II) azurin (λmax = 630 nm). **b** Deconvolution of UV–Vis spectra enables comparison of Cu(I)(*aq*) and Cu(II) azurin concentrations after reaction with Ag ENM of various sizes. Control spectra for BCA assays and sample deconvoluted spectra are given in Additional file 1: Figure S2

Reduction of Cu(II) azurin was assessed with bicinchoninic acid (BCA) as a colorimetric indicator of Cu(I)(*aq*) (Fig. 2). The Cu(I)-BCA absorption peak appears as a shoulder on the Cu(II)-thiol ligand to metal charge transfer (LMCT) band of Cu(II) azurin. As expected, samples with the strongest Cu(I)-BCA shoulder have smaller LMCT bands from Cu(II) azurin, indicative of reduction or loss of Cu(II) from azurin. Control studies demonstrate that the BCA was reactive only with Cu(I)(*aq*), but not with azurin or Ag(I)(*aq*) alone (Additional file 1: Figure S3). In addition, 10-nm citrate-coated Au ENMs reacted with Cu(II) azurin show no evidence of Cu(I) and ESI–MS analysis of the resulting azurin did not reveal any apo- or Au- azurin (data not shown). Consistent with previous reports [21] that the very strong Cu(II)-thiol bond must be reduced before copper release, these results confirm both ENM and redox activity are necessary for Cu(II) azurin-ENM reactivity.

Electronic absorbance spectra were deconvoluted (Additional file 1: Figure S3.b) and spectral contributions were used to calculate the respective concentrations of Cu(I)(*aq*) and Cu(II) azurin (Fig. 2b). Monovalent copper concentrations were uniformly low at 5 µM or less. Consistent with oxidative dissolution results to form Ag(I)(*aq*), no size-dependence was observed in formation of Cu(I)(*aq*), but 10 nm Ag ENMs did react to form the most Cu(I). Concentrations of Cu(II) azurin decreased in accordance with Cu(I)(*aq*) formation; 10 nm Ag ENMs resulted in the lowest amount of Cu(II) in azurin.

Notably, measured total concentrations of copper from Cu(I)(*aq*) and Cu(II) azurin were consistent across samples, at 21.1 ± 1.2 µM. This concentration is 25–30 % lower in than the starting concentration of copper in azurin and has been confirmed by ICP-MS. The remaining copper may be bound to Ag ENMs and Ag ENM-azurin complexes pelleted out during sample processing. These results suggest a small percentage of released copper or Cu azurin may be adsorbing to the ENM surface; a conclusion consistent with evidence of strong azurin–Ag ENM complex formation previously reported [20] and SDS PAGE results indicating azurin is in the hard corona. The calculated Cu(I)(*aq*) concentrations from deconvoluted electronic spectra do not follow an Ag ENM size trend. This may be due to the loss of copper during sample processing, but it may also be explained by variations in Ag ENM curvature with size that alter protein-Ag ENM interactions.

### Conclusions

**Fig. 3:**
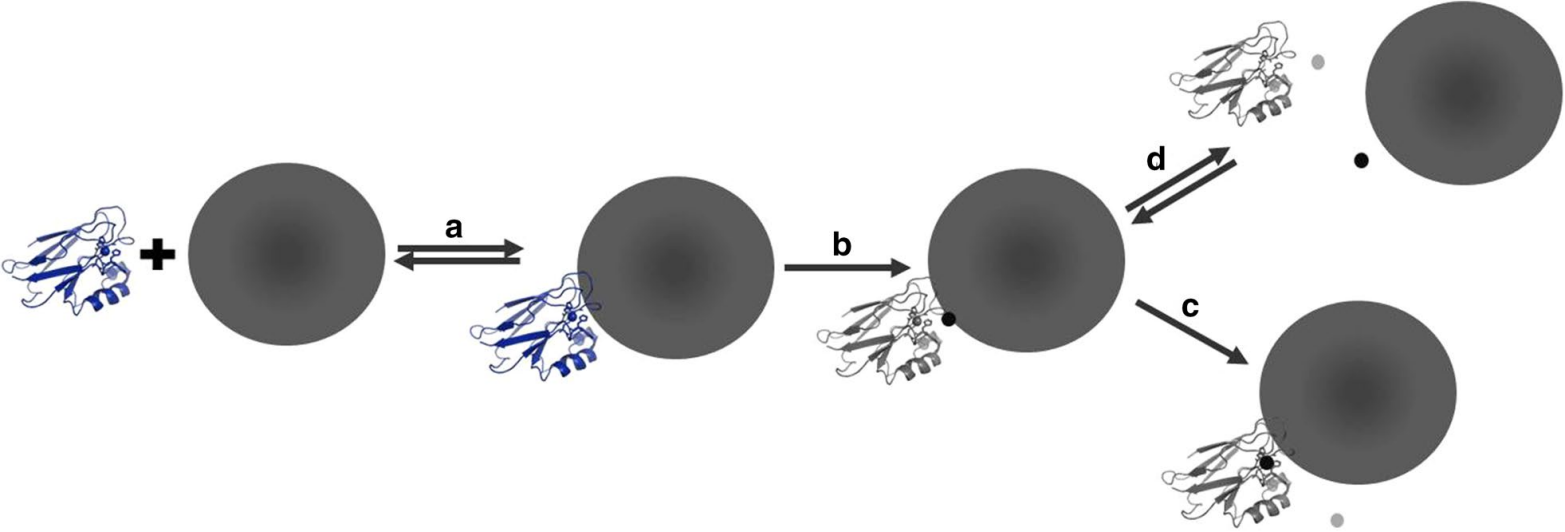
Schematic for proposed Cu(II) azurin–Ag ENM reaction mechanism. **a** Cu(II) azurin (blue protein with Cu(II) shown as *blue sphere* in active site) and Ag ENM (*large grey sphere*) bind to form a complex. **b** Redox reaction oxidizes Ag ENM surface to form Ag(I) (*black sphere*) and reduces Cu(II) azurin to Cu(I) azurin (*light grey* protein with Cu(I) azurin shown with a *light grey* sphere in active site). Weakly bound Cu(I) is displaced from the active site via one of two mechanisms, either via **c** direct displacement to form Ag(I) azurin and dissolved Cu(I)(*aq*) or via **d** two dissolution equilibria to form apo-azurin with dissolved Cu(I)(*aq*) and Ag ENMs with dissolved Ag(I)(*aq*)

Evidence of a protein-Ag ENM redox reaction is twofold. First, Cu(II) azurin increases Ag ENM oxidative dissolution by an order of magnitude over other model redox proteins, even in the presence of a complex mixture of proteins within the corona. Second, Ag ENMs, not Ag(I)(*aq*) or Au ENMs, reduce and displace Cu(II) from azurin. We propose a mechanism of Cu(II) azurin–Ag ENM interactions wherein complex formation results in oxidation of surface Ag on the ENM and reduction of Cu(II) in azurin (Fig. 3). After redox, the Cu(I) is displaced from azurin to either form apo-azurin, or to bind the Ag(I) and form Ag(I) azurin. Either way, the fate of both reactants is altered by increasing Ag ENM dissolution and disrupting the physiological function of the Cu metalloprotein. This mechanism provides a biochemical explanation for Cu(II) azurin reactivity with Ag ENMs, but not with Ag(I)(*aq*) or when Ag ENM-protein complex formation is prevented [20].

This work provides an example of an adventitious protein-ENM redox reaction that alters both metal ENM and protein reactivity. Although many consider dissolution the main mechanism of silver ENM toxicity [14, 24, 25], few have considered the role of the protein corona in redox activity of ENMs and potential role in metal homeostasis [17]. More broadly, this work emphasizes the need to further study adventitious protein redox reactivity with ENMs, especially considering the enzyme-like reactivity of some ENMs, and broadly demonstrates the chemical reactivity of the protein corona at the ENM surface.

## Additional file

10.1186/s12951-016-0160-6 **Additional methods**. SPE extraction and SDS PAGE gels. **Figure S1**. ICP-MS quantification of Ag(I)(*aq*) concentrations from oxidative dissolution of Ag ENMs. **Figure S2.a.** Control UV–Vis spectra for BCA detection of Cu(I)(*aq*). **Figure S2.b.** Sample spectra showing deconvolution of UV–Vis spectra. **Figure S3.** SDS PAGE gels of the protein corona of Ag ENMs.

## Abbreviations

ENMs: engineered nanomaterials
BCA: bicinchoninic acid
ICP-MS: inductively coupled plasma mass spectrometry
SPE: soluble protein extract from *E. coli*
SDS PAGE: sodium dodecyl sulfate polyacrylamide gel electrophoresis
LMCT: ligand metal charge transfer
cyt c: cytochrome c
HRP: horse radish peroxidase
