# Supplementary material for: Beyond the passive interactions at the nano-bio interface: evidence of Cu metalloprotein-driven oxidative dissolution of silver nanoparticles
Source: J Nanobiotechnology. 2016 Jan 22;14:7. doi: 10.1186/s12951-016-0160-6 (PMC4722631; doi:10.1186/s12951-016-0160-6)
Supplement: Supplementary file 1 — 10.1186/s12951-016-0160-6 Additional methods. SPE extraction and SDS PAGE gels. Figure S1. ICP-MS quantification of Ag(I)(aq) concentrations from oxidative dissolution of Ag ENMs. Figure S2.a. Control UV–Vis spectra for BCA detection of Cu(I)(aq). Figure S2.b. Sample spectra showing deconvolution of UV–Vis spectra. Figure S3. SDS PAGE gels of the protein corona of Ag ENMs. [file 12951_2016_160_MOESM1_ESM.doc]

**SUPPLEMENTAL MATERIALS**

SPE Extraction

*E. Coli* was grown in luria broth to an optical density (measured at 600 nm) of 0.5, then spun down (10 min, 21130 rcf) to pellet the cells. The cells were then washed with ice-cold water before being centrifuged again (21130 rcf) and resuspended in 50 mM ammonium bicarbonate (AmBic) buffer. This solution was added drop-wise to liquid nitrogen and then crushed with a mortar and pestle into a fine powder. Then, 50 mM AmBic containing protease inhibitor (Halt Protease Inhibitor Cocktail, purchased from ThermoFisher) was added. The sample was centrifuged for 10 min (3901 rcf, Allegra centrifuge); the pelleted cell debris was discarded and the soluble proteins in the supernatant were kept. The supernatant, now known as SPE, was dialyzed into the buffer of choice (3500 MWCO) before a BCA assay was performed to determine final protein concentration.

SDS PAGE Gels

To visualize the proteins, Ag ENMs and bound proteins were separated by centrifugation (as detailed in procedure for ICP-MS). The ENM pellet was washed 2x with water to remove loosely bound proteins. Then, the pellet was taken up into SDS sample buffer and visualized on a Mini-PROTEAN TGX Precast SDS/PAGE gel (Biorad).

SUPPLEMENTAL FIGURES


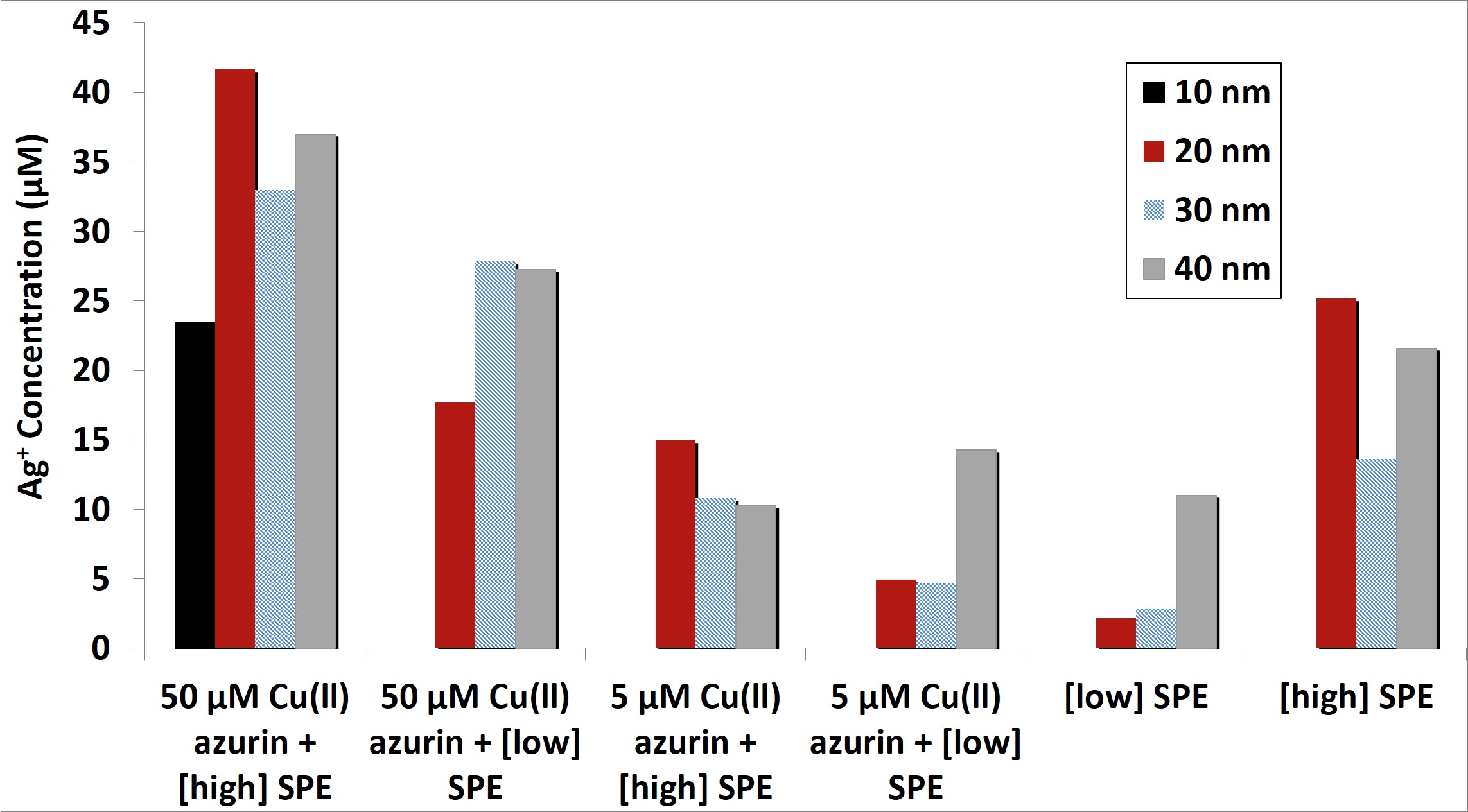


**Figure s1. ICP-MS quantification of Ag(I)(*aq*) concentrations from oxidative dissolution of Ag ENMs.** Ag ENMs was compared across four sizes, 10 (*black*), 20 (*red*), 30 (*blue*), and 40 nm (*grey*). Dissolution of Ag ENMs alone (*labeled control*) is compared to dissolution in the presence of 0.7 and 0.07 mg/ml SPE, as well as SPE with 5 and 50 µM Cu(II) azurin. Silver concentrations from SPE alone has been subtracted from the samples of SPE and azurin for **Figure 1**.


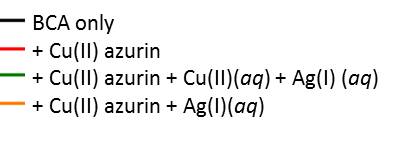


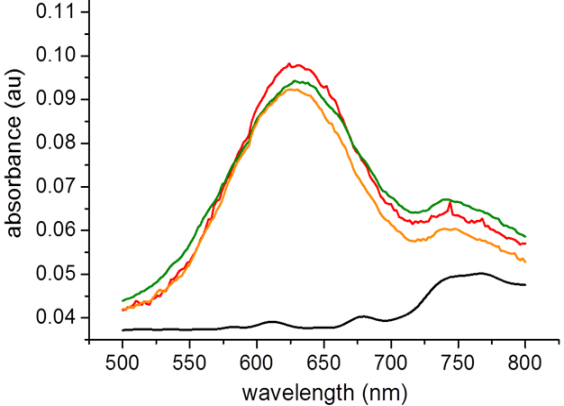


**Figure s2.a. Control UV-Vis spectra for BCA detection of Cu(I)(*aq*).** Control spectra for BCA analysis included UV Vis spectra of 1 mM BCA with the following reactants: 100 μM azurin; 100 μM azurin and 200 μM CuSO4; 100 μM azurin, 200 μM CuSO_4_, and 200 μM AgNO_3_; 100 μM azurin and 200 μM AgNO_3_; and 100 μM AgNO_3_. None of these combinations yielded any increased absorbance or resulted in a peak at 562 nm when spectra were taken within 2 min of BCA addition. Sample control spectra are shown for BCA alone [*black*], and BCA with Cu(II) azurin [*red*], with Cu(II) azurin and dissolved Cu(II) and Ag(I) [*green*], and with Cu(II) azurin and dissolved Ag(I) [*orange*].


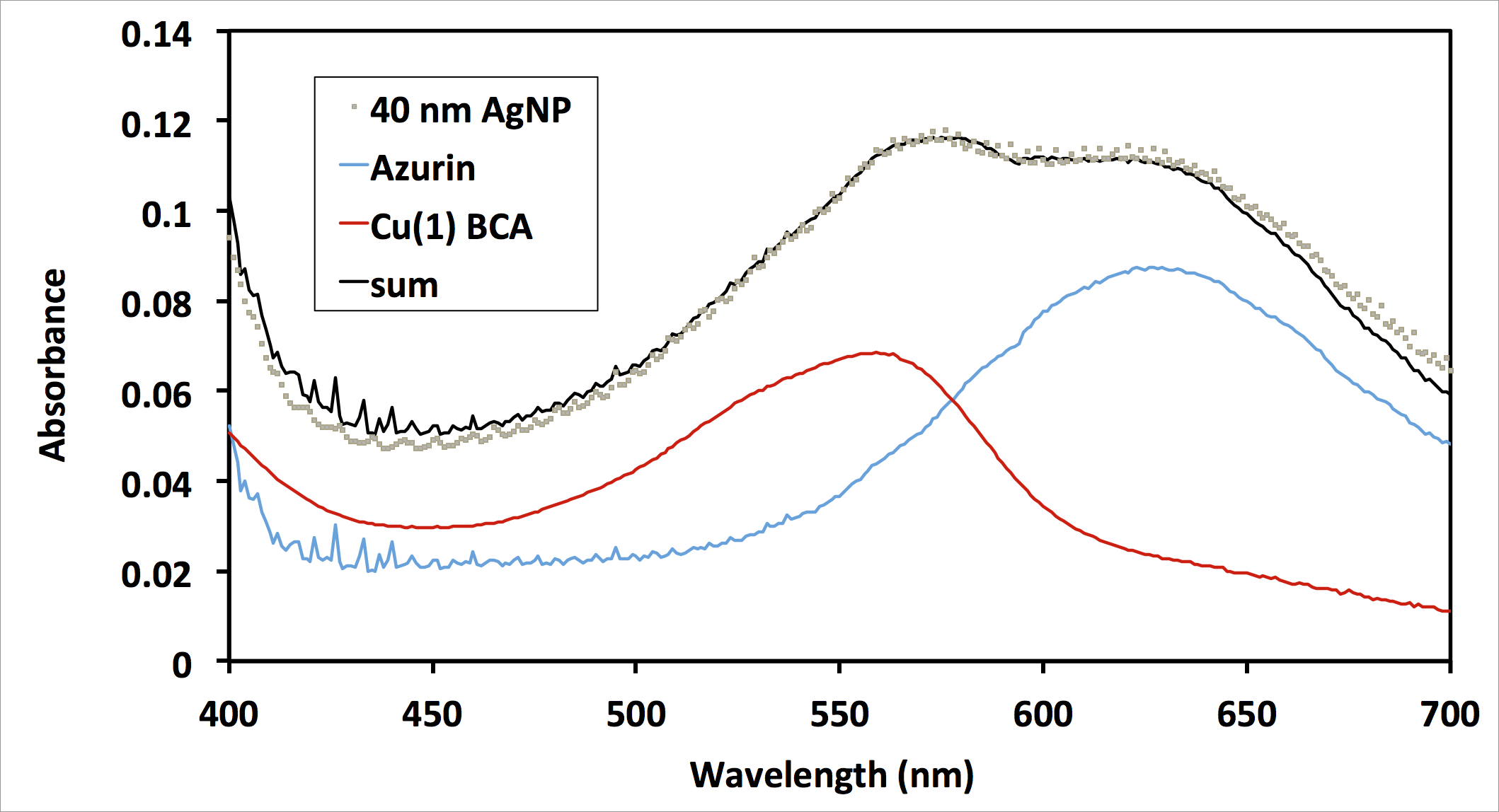


**Figure s2.b. Sample spectra showing deconvolution of UV-Vis spectra.** The BCA-Cu(I) complex (λmax = 562 nm) presents as a shoulder on the Cu(II)-thiol ligand metal charge transfer band from Cu(II) azurin (λmax = 630 nm). The sample deconvolution spectrum for BCA assessment of Cu(I) concentrations in solutions of Cu(II) azurin reacted with 40 nm Ag ENM. The experimental spectrum [light grey] is overlayed with the calculated contributions from the Cu(I)-BCA complex [*red*] and Cu(II) azurin [*blue*], with a calculated sum spectrum [*black*]. Deconvolution was performed using pure spectra, weighting each component to minimize R^2^.


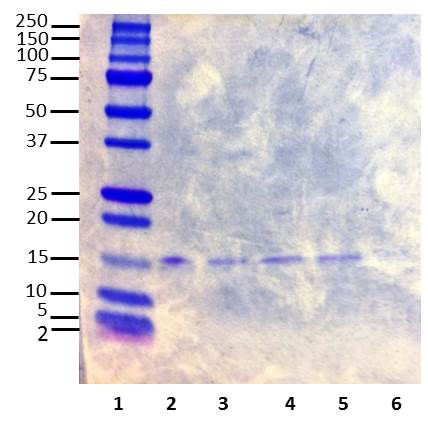

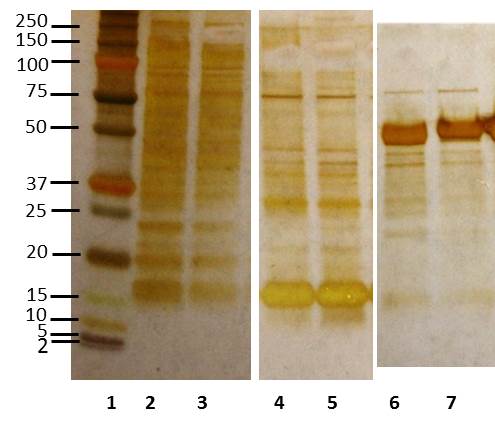


**Figure s3. SDS PAGE gels of the protein corona of Ag ENMs. (a, left)** Coomassie stained gels of the Ag ENM bound proteins when ENMs reacted with 50 µM azurin. Lanes are as follows: (1) protein molecular weight standards, (2) bound protein on 10 nm ENMs, (3) bound protein on 20 nm ENMs, (4) bound protein on 30 nm ENMs, (5) bound protein on 40 nm ENMs, and (6) a sample of just azurin without ENMs as a control to ensure only ENM bound proteins were visualized on the gel. Importantly, Cu(II) azurin (~14 kD) binds consistently across all ENM containing samples, but is not present in the final lane without ENMs. **(b, right)** Silver stained gels of 10 and 20 nm Ag ENM bound proteins when ENMs reacted with SPE mixed with 50 µM proteins of choice. Lanes are as follows: (1) protein molecular weight standards, (2) bound protein on 10 nm ENMs after reaction with 50 µM azurin + 0.7 mg/ml SPE, (3) bound protein on 20 nm ENMs after reaction with 50 µM azurin + 0.7 mg/ml SPE, (4) bound protein on 10 nm ENMs after reaction with 50 µM cyt c + 0.7 mg/ml SPE, (5) bound protein on 20 nm ENMs after reaction with 50 µM cyt c + 0.7 mg/ml SPE, (6) bound protein on 10 nm ENMs after reaction with 50 µM HRP + 0.7 mg/ml SPE, and (2) bound protein on 20 nm ENMs after reaction with 50 µM HRP + 0.7 mg/ml SPE. The large band at ~14 kD in lanes 2 and 3 is presumably azurin; similarly, the large bands at ~12 kD in lanes 4 and 5, and the large band at ~44 kD in lanes 6 and 7 are presumably cyt c and HRP, respectively. Although the same samples with 30 and 40 nm Ag ENMs were also run on SDS PAGE gels and silver stained, they are not shown here because they gave identical results.
